# Ongoing behavioral state information signaled in the lateral habenula guides choice flexibility in freely moving rats

**DOI:** 10.3389/fnbeh.2015.00295

**Published:** 2015-11-04

**Authors:** Phillip M. Baker, Sujean E. Oh, Kevan S. Kidder, Sheri J. Y. Mizumori

**Affiliations:** Department of Psychology, University of WashingtonSeattle, WA, USA

**Keywords:** cognitive flexibility, serotonin, dopamine, lateral habenula, reversal learning, spatial navigation, learning and memory

## Abstract

The lateral habenula (LHb) plays a role in a wide variety of behaviors ranging from maternal care, to sleep, to various forms of cognition. One prominent theory with ample supporting evidence is that the LHb serves to relay basal ganglia and limbic signals about negative outcomes to midbrain monoaminergic systems. This makes it likely that the LHb is critically involved in behavioral flexibility as all of these systems have been shown to contribute when flexible behavior is required. Behavioral flexibility is commonly examined across species and is impaired in various neuropsychiatric conditions including autism, depression, addiction, and schizophrenia; conditions in which the LHb is thought to play a role. Therefore, a thorough examination of the role of the LHb in behavioral flexibility serves multiple functions including understanding possible connections with neuropsychiatric illnesses and additional insight into its role in cognition in general. Here, we assess the LHb’s role in behavioral flexibility through comparisons of the roles its afferent and efferent pathways are known to play. Additionally, we provide new evidence supporting the LHb contributions to behavioral flexibility through organization of specific goal directed actions under cognitively demanding conditions. Specifically, in the first experiment, a majority of neurons recorded from the LHb were found to correlate with velocity on a spatial navigation task and did not change significantly when reward outcomes were manipulated. Additionally, measurements of local field potential (LFP) in the theta band revealed significant changes in power relative to velocity and reward location. In a second set of experiments, inactivation of the LHb with the gamma-aminobutyric acid (GABA) agonists baclofen and muscimol led to an impairment in a spatial/response based repeated probabilistic reversal learning task. Control experiments revealed that this impairment was likely due to the demands of repeated switching behaviors as rats were unimpaired on initial discrimination acquisition or retention of probabilistic learning. Taken together, these novel findings compliment other work discussed supporting a role for the LHb in action selection when cognitive or emotional demands are increased. Finally, we discuss future mechanisms by which a superior understanding of the LHb can be obtained through additional examination of behavioral flexibility tasks.

## Introduction

Multiple decades of research have led to an understanding of many brain areas involved in the ability to switch ongoing behaviors when contingencies change. Changes in behavior can range from a reversal of appetitive or aversive responses, to the adaptation of behavior following subtle environmental cues such as changing seasons. With this range of behavioral flexibility required in complex organisms, it is not surprising that multiple neural systems participate in one or a number of types of related behaviors. In general, behavioral flexibility requires a complex series of neural processes including recognizing environmental cues as well as the internal state of the animal, choosing an appropriate response based on this information, and analyzing the outcome of that choice based on previous expectations in order to plan future behavior. While it is evident that individual forebrain and midbrain systems uniquely control specific functions that enable behavioral flexibility (e.g., outcome analysis or action selection) or determine the current type of behavioral flexibility, e.g., reversal learning vs. set-shifting, other systems appear to play more general roles across many forms of adaptive behavior. Among these are two monoamine neurotransmitter systems: the dopamine (DA) and serotonin (5-HT) systems.

The DA system has been implicated in nearly all aspects of behavioral flexibility performance from action selection to recognizing a change in outcomes (Spirduso et al., 1985; Barnéoud et al., 2000; Ragozzino, 2002; Lee et al., 2007; De Steno and Schmauss, 2009; Kehagia et al., 2010). For example, striatal DA is required for the initiation of motivated actions as selective dopaminergic lesions to the median forebrain bundle results in impaired memory for learned motor programs which is interpreted as impaired top-down movement control (Ridley et al., 2006). DA release within the striatum is also observed when animals experience reward predictive cues or unexpected changes in reward expectations which is thought to relate to motivational aspects of rewarding actions (Stuber et al., 2008; Wassum et al., 2012; Volman et al., 2013). Additionally, prefrontal DA release is selectively increased on the reversal but not repeated performance of a spatial reversal learning task (van der Meulen et al., 2007). 5-HT contributes to behavioral flexibility tasks in a complimentary manner to DA through tracking of expectation for behavioral flexibility. For example, neural activity in the dorsal raphe (DRN; which includes many forebrain projecting 5-HT neurons) tracks ongoing behaviors in relation to upcoming outcomes (Bromberg-Martin et al., 2010; Inaba et al., 2013; Liu et al., 2014). Decreasing 5-HT availability through either excitotoxic lesions or tryptophan depletion impairs behavioral flexibility while increasing it with selective serotonin reuptake inhibitors (SSRIs) enhances it (Bari et al., 2010; Brown et al., 2012; Izquierdo et al., 2012; Wallace et al., 2014). This has led many to suggest that 5-HT signaling contributes to reward or aversive learning especially when behavioral expectancy signals must be updated for future behavioral choices such as when risk is involved in decision making (Doya, 2008; Robbins and Arnsten, 2009; Bari et al., 2010; Liu et al., 2014). Additionally, Groman et al. (2013) provided evidence that these systems interact with one another in a complimentary fashion during behavioral flexibility such that a balanced increase between 5-HT and DA levels in the orbitofrontal cortex and striatum is correlated with ideal reversal learning performance.

Despite well-established roles for monoamine systems in behavioral flexibility, it is not well understood how forebrain structures that are involved in behavioral flexibility influence the DA and 5-HT systems. There is growing interest in understanding how information is processed by structures which influence the DA and 5-HT systems. However, this is a difficult problem since, in order to contribute to a wide variety of behaviors, these systems must receive information about the current behavioral and emotional state of an animal in order to organize and reinforce beneficial behaviors. One key structure that is poised to relay both current behavioral and emotional/internal state information is the lateral habenula (LHb) which can influence both the DA and 5-HT systems via direct and indirect connections (Lecourtier et al., 2008; Goncalves et al., 2012; Sego et al., 2014). It has been suggested that the LHb possess two separate streams of information comprising the medial and lateral portions of the LHb (Hikosaka, 2010; Proulx et al., 2014). Neural recording studies in animals performing complex behavioral tasks that require behavioral flexibility has yet to resolve whether different regions of the LHb respond to different aspects of behavior. Below we describe, and then test, a possible role for the LHb in regulating DA and/or 5HT modulation when animals must flexibly switch ongoing behaviors as contingencies change.

Many potential roles for the LHb in behavior have been proposed based on the diverse effects observed during either neural recording or after experimental manipulation. Early reports of the behavioral role of the LHb included a currently not well defined role in olfactory processing, as well as mating behavior, and aversive or reward learning (Sutherland, 1982). More recent work has focused on the role of the LHb in aversive responses. One such proposal which has gained prominence as of late is a role in inhibiting DA neurons in response to aversive outcomes or predictions during Pavlovian learning (Hikosaka, 2010; Proulx et al., 2014). The role of the LHb is less well understood in goal directed behaviors that rely on behavioral flexibility to obtain a desired outcome. Recent reports and new data presented below support our hypothesis that the LHb plays a role in the execution of specific goal directed actions when the use of complex strategies, or switching of strategies, is required. Specifically *we propose that LHb signals to brainstem monoamine systems information about the ongoing behavioral state of an animal for the purpose of organizing adaptive actions aimed at receiving rewards or avoiding punishment*. Based on data presented within, it is likely that at least in the rat, this information about behavioral states broadcast to both the serotonergic and dopaminergic systems to then further be integrated with additional input distinct to each system. This view is supported by the role that both DA and 5-HT are known to play in both goal directed activity in general as well as behavioral flexibility specifically. The aim of this review is to synthesize a diverse body of research aimed at understanding how the LHb functions when animals are required to change ongoing or innate actions in order to receive reward or avoid punishment. Our interpretation will emphasize the known role of afferent and efferent structures of the LHb and how they inform the role this structure plays in behavioral flexibility. Additionally, preliminary experiments in our own lab are discussed in relation to this hypothesis of LHb function. Finally, novel means of testing this hypothesis are discussed in relation to state of the art techniques now available to dissect circuit function.

## Functional Anatomy of the Lateral Habenula (LHb)

The LHb can be divided into as many as 10 subregions based on either streams of input and output or identities of neuronal protein expression (Andres et al., 1999; Geisler et al., 2003; Aizawa et al., 2012; Wagner et al., 2014). Due to the already relatively small size of the habenular complex itself, however, it is often treated somewhat more homogeneously given the practical challenges in isolating such small subdivisions. Recent advances in promoter driven Cre mice lines however, offer a way forward in addressing subregion specific contributions to LHb function at a basic level. The LHb is often divided into a medial segment and a lateral segment based on the targets of projection neurons: the medial portion mainly targets the median and DRN and the lateral portion largely projects to the rostromedial tegmental nucleus (RMTg; Kim and Chang, 2005; Proulx et al., 2014). It is worth noting however, that this division is based mainly on interest in the LHb control of monoamine structures, particularly in cognition, and this to some extent has minimized our appreciation of the prominent projections to the posterior hypothalamus, dorsal tegmentum and periaqueductal gray identified in both rats and mice (Araki et al., 1988; Quina et al., 2015). These latter projections are also largely segregated although somewhat overlapping (Harris et al., 2014). It is promising for future studies that the overall cytoarchitecture and circuitry appears similar between mice and rats (Geisler et al., 2003; Goncalves et al., 2012; Wagner et al., 2014; Quina et al., 2015) although some differences between rodents and primates have been observed (Parent et al., 1981; Araki et al., 1984; Hong and Hikosaka, 2008).

### LHb Afferent Structures and their Role in Behavioral Flexibility

**Figure 1:**
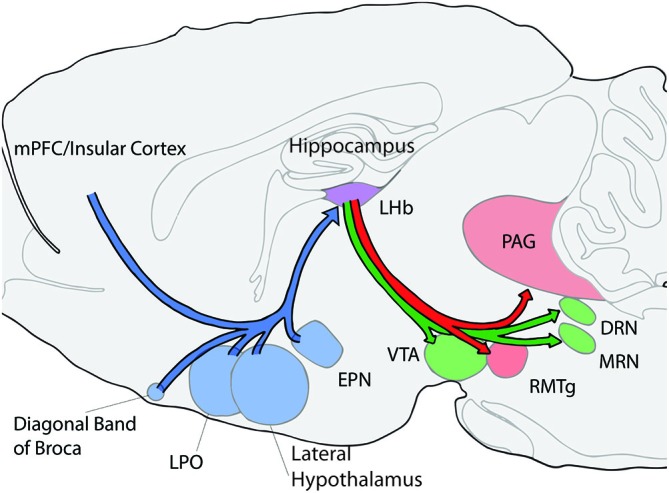
**Schematic of selected afferent and efferent connections of the lateral habenula (LHb; shown in purple).** Afferent connections/structures are shown in blue, efferent connections are shown in red, and bidirectional connections are in green. LHb, lateral habenula; PAG, periaqueductal gray; DRN, dorsal rahpe; MRN, median raphe; RMTg, rostromedial tegmental nucleus; VTA, ventral tegmental area; LPO, lateral preoptic area; EPN, entopeduncular nucleus; and mPFC, medial prefrontal cortex.

Insight into the potential types of functions that the LHb contributes may be obtained by understanding the roles that LHb afferent systems play in behavioral flexibility (Figure 1). The LHb receives input from many different brain areas which are often divided into several major categories, the basal ganglia, the hypothalamic areas, and the limbic cortical systems (Sutherland, 1982; Lecourtier and Kelly, 2007; Hikosaka, 2010). The LHb also receives DA and 5-HT input from the ventral tegmental area and the median raphe (MRN) making these connections with monoaminergic systems symmetrical (Beckstead et al., 1979; Skagerberg et al., 1984; Vertes et al., 1999). Overall, the patterns of connectivity of the LHb raise many possibilities for its involvement in a wide variety of behavioral flexibility functions. For a more complete view of all afferent and efferent connections of the LHb see (Lecourtier and Kelly, 2007) or (Quina et al., 2015).

The main input from the *basal ganglia* arises from the entopeduncular nucleus (EPN; Nagy et al., 1978; Araki et al., 1984). In rodents a majority of EPN fibers, especially in the rostral portion of the nucleus project to the entirety of the LHb (Parent et al., 1981; Araki et al., 1984; Vincent and Brown, 1986). In monkeys this projection appears to originate from a unique and restricted region of the internal globus pallidus (GPi) mainly from the dorsal and ventral boarders (Parent et al., 1981; Hong and Hikosaka, 2008). It is not well understood why this species difference exists. Interestingly, in rats neurons from the EPN projecting to the LHb contain both glutamate and GABA (Araki et al., 1984; Shabel et al., 2014).

The basal ganglia plays a prominent role in behavioral flexibility ranging from response reversal learning, to inhibiting ongoing actions, to switching foraging patches when resources become scarce (Schwartzbaum and Donovick, 1968; Seamans and Phillips, 1994; Hills, 2006; Bryden et al., 2012). However, less is known about the role of the most immediate structure projecting to the LHb, the EPN. In monkeys, neurons recorded from the GPi respond to reward consumption, reward predictive cues or probabilities, and exploratory behavior (Hong and Hikosaka, 2008; Joshua et al., 2009). In rodents however, to date only one paper has examined firing properties of EPN neurons in freely moving animals (Benhamou and Cohen, 2014). This study found that a majority of cells had lower firing rates similar to those observed in monkey GPi neurons which project to the LHb when exploring an open field. The proportion of cells in this group roughly matches proportions of LHb projecting neurons in the EPN (~66%; van der Kooy and Carter, 1981). This could mean similar functions are served by these EPN neurons in rats as is observed with monkeys in relation to goal directed behavior, however, it remains a speculation at this point (Benhamou and Cohen, 2014). In terms of understanding behavioral flexibility, it is difficult to isolate a role for the EPN in rats due to the lack of *in vivo* electrophysiological studies as well as confounds with motor effects and sensory motor integration observed following experimental manipulation (Dacey and Grossman, 1977; Scheel-Krüger et al., 1981; Sarkisov et al., 2003; Schwabe et al., 2009). However, these confounds can be controlled for by unilateral or sequential lesion techniques, especially those that use fiber sparing methods (Lutjens et al., 2011). Using these methods, it is known that the EPN contributes to active avoidance operant behavior when animals are given periods of safe pressing for reward intermixed with times when a cue indicates that presses result in shock. EPN lesions result in continued pressing during the shock period (Margules, 1971; Chavez-Martinez et al., 1987). Accurate performance on this task requires the integration of emotional information (shock avoidance) with appetitive signals (hunger) raising the possibility that a downstream target of the EPN such as the LHb may also be involved in the integration of emotional and motivational information especially when one considers LHb connections with the limbic system.

Another major projection to the LHb originates in the *hypothalamus* including the lateral preoptic area (LPOA) and the lateral hypothalamus (Herkenham and Nauta, 1977; Parent et al., 1981). These hypothalamic areas are known for their role in emotional arousal, cue associations and feeding behavior (Stratford and Wirtshafter, 2012; Sohn et al., 2013; Cole et al., 2015). The lateral hypothalamus has also been connected with attention and learning based on cues for both positive and negative outcomes (Ono et al., 1986). Further, orexin/hypocreatin and melanin-concentrating hormone neurons originating in the lateral hypothalamus likely project to the LHb as staining for these receptors or compounds is found in the LHb (Skofitsch et al., 1985; Peyron et al., 1998). This is interesting in relation to behavioral flexibility as the lateral hypothalamus orexin neurons are proposed to be critical for a flexible arousal system in the brain (Kosse and Burdakov, 2014). Melanin concentrating hormone also participates in feeding behaviors as well as emotional regulation and stress (Hervieu, 2003; Saito and Nagasaki, 2008). It is not known to date whether the LHb also contributes to these behaviors. An additional potential contributor to LHb in behavioral flexibility is the LPOA. Several studies have reported that neurons within the LPOA responded to cues that predicted either positive or negative outcomes similarly, suggesting a role in attention or arousal as would be required during behavioral flexibility (Linseman, 1974; Ono and Nakamura, 1985). Based on the behaviors in which both major LHb afferent systems projections, the EPN and hypothalamic areas, are involved, the LHb stands in an ideal position to integrate sensory/motor, reward, arousal, and emotion/stress related information to guide behavior as both the internal and external states of the animal change. This integrated signal can then be relayed to midbrain areas coherently and quickly.

Identified projections from *frontal cortical areas* to the LHb also support LHb involvement in behavioral flexibility (Greatrex and Phillipson, 1982; Kim and Lee, 2012). Projections from the prelimbic and infralimbic regions of the mPFC are largely confined to the medial portions of the LHb while the anterior cingulate cortex (ACC) and insular cortex project to more lateral areas (Kim and Lee, 2012). The medial prefrontal cortex (mPFC) is known to be important when established behavioral strategies must be overridden as is required in a number of behavioral flexibility tasks (Seamans et al., 1995; Dalley et al., 2004; Ragozzino, 2007; Shaw et al., 2013). Typically, these mPFC deficits manifest as perseverations on the previous reward contingencies which are interpreted as an inability to inhibit the previously relevant behavior (Dias and Aggleton, 2000; Ragozzino et al., 2003). In contrast, the ACC contributes to general discrimination learning mechanisms as both its lesion or temporary inactivation result in non-specific error patterns and delayed learning (Dias and Aggleton, 2000; Ragozzino and Rozman, 2007; Kosaki and Watanabe, 2012). Neurons recorded from the ACC show greater activation with higher task demands supporting its role in difficult tasks that require switching behaviors (Johnston et al., 2007). Cognitively demanding tasks are known to result in sustained tonic DA signaling (Abercrombie et al., 1989; Phillips et al., 2004). Thus input from the ACC and mPFC likely influence the role of the LHb in controlling monoamine projections discussed below. Specifically, we propose that prefrontal information about behavioral context and task difficultly are relayed to the LHb where they become integrated with other input such as reward and effort (from basal ganglia) to influence monoamine resources. This possibility is supported by a study which showed that LHb inhibition or excitation *in vivo* resulted in regionally specific changes in tonic dopamine levels (Lecourtier et al., 2008).

### Efferent Connections of the LHb to the DA and 5-HT Systems and their Role in Behavioral Flexibility

The dopaminergic system has been connected with behavioral flexibility and reinforcement learning for many years (Roberge et al., 1980; Schultz, 1998; Heyser et al., 2000; Kehagia et al., 2010). The DA system is mainly contained within two areas, the substantia nigra pars compacta (SNc) and the ventral tegmental nucleus (VTA). The LHb can strongly influence DA neurons in both areas as even a single LHb electrical stimulation pulse can inhibit DA firing in both structures as for as long as 250 ms (Christoph et al., 1986). It is thought that this effect is due to both direct excitatory projections onto GABAergic interneurons as well as indirect projections via the RMTg (Brinschwitz et al., 2010; Balcita-Pedicino et al., 2011; Goncalves et al., 2012). Both the VTA and SNc project to numerous limbic system and cortical areas that influence behavioral flexibility (Fallon, 1981; Swanson, 1982; Oades and Halliday, 1987). A number of excellent reviews are available on this topic (Floresco and Magyar, 2006; Kehagia et al., 2010; Klanker et al., 2013), and so only a selected number of studies will be highlighted here. Historically, depletion of DA projections to the forebrain was found to cause a reduction in the ability to initiate goal directed actions. However, reflexes and automatic motor movement remained undisturbed suggesting that DA plays a critical role in goal directed actions via the forebrain. These findings were commonly related to Parkinson’s Disease as a gross depletion of DA is a hallmark of that condition. However, in addition to the motor symptoms of Parkinson’s, deficits in behavioral flexibility are also common. This led to interest in DA contributions to behavioral flexibility.

In general, DA neurons themselves, both within the VTA and SNc, respond to reward predictive cues or reward/punishment (Schultz, 1998; Matsumoto and Hikosaka, 2009). In addition, several basal ganglia areas require DA input in order for animals to successfully enact a number of behavioral flexibility tasks. Nucleus accumbens depletion of DA using the neurotoxin 6-OHDA impairs spontaneous exploratory behavior, discrimination learning and reversal learning (Taghzouti et al., 1985). In contrast, DA depletion in the dorsomedial striatum had only a minor effect on reversal learning as evidenced by slight increases in the magnitude of difference between lesioned and sham animals (O’Neill and Brown, 2007). However, more substantial lesions of mouse dorsal striatum have been found to impair rule switches from a turn to a cue based strategy on a water-based U-shaped maze (Darvas et al., 2014). Using *in vivo* cyclic voltammetry, striatal DA has been found to signal reward predictive cues and unexpected rewards (Aragona et al., 2009; Brown et al., 2011). Thus the striatum represents a possible node in a network that includes the LHb and the DA system to signal when expected events begin or when expectations are violated and new behaviors must be implemented. Another possible actor in this network is the prefrontal cortex. Medial prefrontal 6-OHDA lesions were found to impair the ability of animals to acquire a response set indicating that DA facilitates set or strategy formation (Crofts et al., 2001). Similarly, using *in vivo* microdialysis it was found that DA levels increased in the mPFC during both the acquisition and switching of a brightness/texture discrimination as well as when reward was given unpredictably (Stefani and Moghaddam, 2006). However, when reward was given on all arm entries in a non-contingent predictable manner, no changes were observed (Stefani and Moghaddam, 2006). Overall, it is likely that the basal ganglia and prefrontal cortex together to signal when behaviors are to be learned or performed and when these learned behaviors must be changed due to changes in reward outcomes.

Recently, there has been a growing interest in how specific firing patterns of DA neurons affect goal directed behavior. Two commonly studied modes of DA transmission are tonic and burst firing. Evidence suggests that burst firing plays a key role in reward prediction and learning in several brain areas (Schultz, 1998; Matsumoto and Hikosaka, 2009; Brown et al., 2011). Tonic firing on the other hand, is thought to influence the plasticity in various circuits (Frank, 2005; Goto and Grace, 2005; Dreyer et al., 2010). One particularly interesting study blocked burst firing in DA cells by selectively removing their N-methyl-D-aspartate (NMDA) receptors. This resulted in a reduced ability to learn and reverse a cue based reward association on a *t*-maze (Zweifel et al., 2009). The LHb may differentially affect tonic and burst firing aspects of DA transmission contributing to unique control over the role of DA in goal directed actions. In support of this hypothesis, inhibition of the LHb in awake and behaving animals resulted in a sustained (~1 h) increase in DA in the prefrontal cortex, nucleus accumbens and the lateral striatum (although with different magnitudes and time courses) suggesting a role in tonic neurotransmission (Lecourtier et al., 2008). Stimulation of the LHb during receipt of a reward was shown to block reward-induced DA neuron excitation and shift preferences to the alternative choice suggesting the LHb also plays a role in burst transmission (Stopper et al., 2014). More studies are needed to determine how the LHb might act on tonic and burst firing modes of DA within the same task.

Analogs to the dopaminergic system, *the serotonergic system* also has a complex role in behavioral flexibility. There are two main 5-HT nuclei in the brain, the DRN and MRN, which together project to a majority of other brain structures (Bobillier et al., 1979; Waterhouse et al., 1986; Sim and Joseph, 1992; Vertes et al., 1999; Vasudeva et al., 2011). Limited research has examined the role of the DRN and MRN themselves in behavioral flexibility. One study indicated that electrolytic lesions of the MRN caused an impairment on an egocentric reversal learning task without affecting initial acquisition (Wirtshafter and Asin, 1986). However, most research has focused on anxiety/depressive-like or ingestive behaviors in relation to MRN functions (Wirtshafter, 2001; Andrade et al., 2013; López Hill et al., 2013; Zangrossi and Graeff, 2014). Likewise there are limited data on the effect of DRN manipulation of behavioral flexibility. Recording from DRN neurons in monkeys has revealed tonic changes in firing rate associated with ongoing goal directed behaviors that continue until after receipt of the reward (Nakamura et al., 2008; Bromberg-Martin et al., 2010). Additionally, electrical stimulation of the DRN can reinforce instrumental behavior (Corbett and Wise, 1979; Rompre and Miliaressis, 1985). More recent work using optogenetic targeting of DRN subpopulations suggests that both 5-HT and non-5-HT mechanisms contribute to reinforcing instrumental behaviors (Liu et al., 2014; McDevitt et al., 2014). These data suggest that the DRN serves to reinforce specific goal directed actions.

While the specific contributions of the MRN and DRN to behavioral flexibility remain relatively unknown, the impact of systemic 5-HT manipulation has been much more extensively examined in behavioral flexibility tasks (Evers et al., 2007; Lapiz-Bluhm et al., 2009; Baker et al., 2011; Mohler et al., 2011; Pennanen et al., 2013; Barlow et al., 2015). The selective 5-HT reuptake inhibitors (SSRI) citalopram or fluoxetine or deletion of the serotonin transporter have been shown to enhance behavioral flexibility selectively when animals are required to switch from either a learned or pre-potent behavior (Brigman et al., 2010; Brown et al., 2012). Additionally, serotonin depletion through tryptophan deprivation has been shown to impair behavioral flexibility in humans (Evers et al., 2007), however, in rats either no deficits have been observed or at higher doses or using parachloroamphetamine, a more fundamental deficit in reinforcement learning has occurred (Masaki et al., 2006; van der Plasse and Feenstra, 2008; Izquierdo et al., 2012). Specific serotonin receptors have also been examined in behavioral flexibility as they are commonly modulated by atypical antipsychotics and thus may offer therapeutic potential to patients who experience deficits in behavioral flexibility. Systemic injection of 5-HT2A or 5-HT2C receptor antagonists have impaired or improved the ability to reverse a spatial strategy respectively, *while systemic injection of a 5-HT2A antagonist* improved switching between visual cue and response guided strategies (Boulougouris et al., 2008; Baker et al., 2011). In addition, 5-HT6 and 5-HT7 receptor antagonists have been shown to improve behavioral flexibility in both control animals and disease models (Mohler et al., 2011; Nikiforuk, 2012; Nikiforuk and Popik, 2013; Wallace et al., 2014). The nature of these 5-HT effects suggests that the 5-HT system, through its various projections and receptors, plays diverse roles in behavioral flexibility depending on the specific conditions of a given experiment. Nonetheless, despite this diversity of action within the 5-HT system, an overriding role in behavioral flexibility is clearly evident which suggests that both general as well as specific forms of input to the 5-HT nuclei may be required during behavioral flexibility tasks.

Overall, common to both the DA and 5-HT system is their well-documented role in goal directed learning both when an animal must initially learn a behavioral discrimination and when a switch in behavior is required. Both types of learning require both information about the internal state of the animal, e.g., motivation, and motor action planning and execution. As demonstrated in the following discussion of LHb manipulations as well as the results of our own research presented herein, the LHb likely informs both monoamine systems of the ongoing or recently chosen relevant behavior so that this information can be used by the DA and 5-HT nuclei to achieve the specific functions ascribed to each.

## A Role for the LHb in Behavioral Flexibility

To our knowledge, no experiments have examined the role of the LHb in behavioral flexibility tasks. However, Matsumoto and Hikosaka (2007) found that LHb neurons tracked reversals in task contingencies. Apart from behavioral flexibility specifically, several lines of evidence support a role for the LHb in ongoing goal directed activity. The sole study looking at LHb neurons while a rat performed a behavior used a pellet chasing task. The authors found that a majority of neurons tracked velocity while the animals performed the task (Sharp et al., 2006). This could be interpreted as support for LHb’s role in tracking goal directed behavior. Early reports of the effects of lesions to the LHb were typically performed using electrolytic lesions and often included damage to the medial habenula and surrounding thalamic nuclei/interpeduncular nucleus. In experiments under these conditions, it was found that rats were unable to switch behaviors or maintain behaviors when contingencies were changed using appetitive rewards (Thornton and Evans, 1984; Thornton and Davies, 1991). Another study by this group revealed an interesting interaction between stress and goal directed activity. Specifically, rats were given a one way active avoidance test in which they were required to climb onto an escape platform in order to avoid a shock. At low shock intensities, no differences were observed between controls and lesioned animals. However, when either the shock intensity was increased or the platform raised, lesioned animals showed a deficit in escape latency (Thornton and Bradbury, 1989). This suggests that when either stress (internal state) or effort (goal directed action) is increased, the LHb is needed for effective behavioral responses. It is unknown to date whether these effects related to stress or effort might also affect performance on more standard tasks of behavioral flexibility.

More recent studies have used fiber sparing lesions or temporary inactivation to restrict the extent of damage to adjacent areas as it was suggested that related damage may have contributed to effects observed in earlier LHb lesion studies (Wilcox et al., 1986; Thornton et al., 1994). Using fiber sparing excitotoxic lesions selectively in the LHb, an effect on hippocampal dependent learning has been observed in both the Morris water maze and in a spatial recognition task (Goutagny et al., 2013; Mathis et al., 2015). It was also found that LHb inactivation using GABA agonists impaired performance on a cue guided version of the water maze after the initial spatial memory test. One possibility is that animals were unable to alter their behavior after performing the previous test as would be expected if the LHb is important for behavioral flexibility (Mathis et al., 2015). At the very least, it supports a role for the LHb in hippocampal dependent spatial memory. Furthermore, Stopper and Floresco (2014) found that inactivation of the LHb was sufficient to disrupt both probability and temporal discounting. This deficit manifested as animals choosing equally either option in the two choice task (i.e., at chance levels), a result that could be interpreted as an inability to reorganize the appropriate behavior based on the specific cues in the environment. This view is in support of the proposed role of the LHb in organizing adaptive actions. Additionally since discounting tasks rely on choices determined by both subjective and objective value, the role of the LHb is not solely to signal punishment but rather LHb seems to have a richer role that includes decisions related to choice preference.

Based on the extant literature, then, one possibility is that when learning is either stressful or requires additional effort, either cognitive or physical, the LHb relays important information from forebrain areas such as the EPN and limbic areas perhaps to guide decision making relevant to adaptive strategic choices. To begin to probe the role of the LHb in behavioral flexibility under cognitively demanding circumstances, we undertook the following set of studies to clarify its important role when animals must switch from an ongoing to a newly relevant strategy.

The first experiment used *in vivo* extracellular recordings to address the role that the LHb plays in both spatial memory and behavioral flexibility when the external environment changes in a number of different ways. This task has been found to elicit reward prediction error (RPE) signals within the VTA of freely behaving rats raising the likelihood that these signals may be found in the LHb during this task as well (Puryear et al., 2010; Jo et al., 2013). Specifically, animals were taught to navigate a radial arm maze in order to collect rewards in which alternating arms had either a large or small reward. In the second half of a test session, the contingencies of the task were changed by switching to darkness, omitting some of the rewards, or reversing the reward contingencies. Three different manipulations were administered in order to probe if the LHb responded differently in a number of behavioral contexts or whether it played a more common role in each version of the task.

A second set of experiments examined the role of the LHb in a repeated probabilistic reversal learning maze task via inactivation with the GABA agonists baclofen and muscimol. In experiment 2a, rats were trained on a *T*-maze to make egocentric or spatial discriminations for 10 consecutive trials after which the contingencies were reversed. The correct arm was rewarded on 80% of the choices while the incorrect arm was never reinforced. This reward schedule was chosen for several reasons. First, it made the task more difficult than a deterministic reversal task causing the animals to commit more errors for analysis. Additionally, because the reward was probabilistic, error patterns could be examined for sensitivity to positive and negative reinforcement following LHb inactivation further revealing the role of the LHb in behavioral flexibility. In order to examine whether the effects observed in experiment 2a were due to general learning or recall effects or rather due to flexible behavior *per se*, a control experiment 2b was carried out in which inactivation of the LHb was administered either during initial acquisition of the probabilistic task, or during recall of the contingency on the following day. All of these experiments in varying ways required animals to be flexible in their behavior, thereby allowing for an examination of how the LHb contributes to this ability in rats.

## Materials and Methods

### Subjects

Twelve male Long-Evans rats (350–500 g, Simonsen Laboratories) and 31 male Long-Evans rats (350–500 g, Charles River) used in experiments 1 and 2a and 2b respectively, were individually housed in a temperature-controlled environment with a 12 h light/dark cycle. All experiments were conducted during the light phase. All subjects were given food and water *ad libitum* and handled for at least 5 days before behavioral testing began. During behavioral testing, rats were maintained at 85–90% of their maximum free feeding body weight. All animal care was conducted according to guidelines established by the National Institutes of Health and approved by the University of Washington’s Institute for Animal Care and Use Committee.

#### Experiment 1: Differential-Reward, Spatial Memory Task

**Figure 2:**
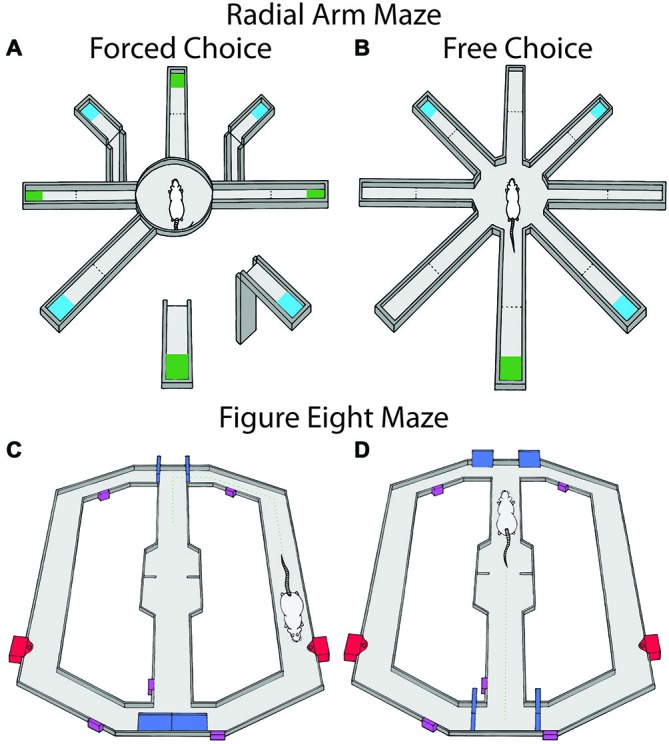
**Maze tasks used in experiments 1 and 2.** Maze arms are raised and lowered remotely. Large reward amounts are represented in blue and small reward amounts are in green and were alternated between arms. **(A)** The radial arm maze during the forced choice portion of the task. Animals were first given four forced choice arms and then were allowed to freely choose for the remaining rewards. **(B)** The free choice portion consisted of both the previously available arms in the forced phase as well as the previously unavailable arms. In a second block of trials, reward contingences were changed by either omission, reversal of contingencies or running in darkness. **(C,D)** show the figure eight maze for probabilistic learning and reversal during the trial start and decision portions of the task respectively. Doors controlling rats’ access to maze compartments are shown in blue, sensors used for the automated program are shown in purple, and the food wells for each arm are represented in red. Rats received reinforcement on 80% of trials for correct choices and 0% for incorrect choices. When rats chose the correct arm 10 trials in a row, contingencies were reversed.

Behavioral training of a differential reward spatial memory task was conducted on an 8-arm radial maze as described previously (Puryear et al., 2010). The black Plexiglas maze consisted of a central platform (19.5 cm dia) that was elevated 79 cm off the ground with eight radially-extending arms (58 × 5.5 cm), see Figure 2A. At the end of the maze arms was a small receptacle that contained, on alternating arms, either a small (0.2 mL) or large (0.6 mL) amounts of “reward” (50% diluted Ensure chocolate milk). Each maze arm was hinged such that access to the rewards were remotely controlled by moving the proximal segment up or down, connecting or disconnecting the ends of arms from the central platform. The maze was surrounded by black curtains with several visual cues for orientation (Figures 2A,B).

Rats habituated to the radial arm maze through free exploration initially with randomly placed puddles of reward, then with rewards only at the end of arms. Once the animals consistently visited the ends of arms, training of the differential reward spatial memory task began. Each session consisted of two blocks of five trials. Each trial consisted of a study phase and a test phase. During the study phase of each trial, four of the eight arms (two large-reward and two small-reward arms) were pseudorandomly selected and presented individually. After presentation of the fourth arm, the test phase began by making all maze arms accessible at once. The rat was required to collect the remaining rewards. Revisits to previously visited end of arms within a trial were coded as errors. When the animal returned to the central platform after visiting all eight arms, the arms were lowered so that the rat was confined to the platform, and the experimenter re-baited the arms. The locations of differentially rewarded arms were held constant for each rat throughout training but were counterbalanced across rats. Once rats made an average of one or fewer errors per trial on a training day, they underwent a surgical procedure for the implantation of recording electrodes. Training ranged from 20–40 sessions across rats.

During recording sessions, Block 1 consisted of four baseline trials, where reward locations were kept identical to that during initial training. One of the three experimental manipulations was conducted during the four trials of block 2: reward switch, reward omission, or darkness. Large and small reward locations were switched during “reward switch”. In “reward omission” trials, two pseudorandomly chosen rewards (one large, one small) were omitted during the study phase. Reward switch and omission creates conditions where the animal would encounter larger than expected rewards, smaller than expected rewards, and unexpected absence of rewards. In the darkness condition, maze lights were turned off to eliminate access to visual cues. On average, rats were exposed to 10 switch and omission sessions and seven darkness sessions.

#### Experiments 2a and 2b: Repeated Probabilistic Reversal Learning

Eleven rats were trained on a modified *T*-maze with return arms so that rats could freely return to the start location after rewards were collected (Figures 2C,D). The maze was controlled by custom built robotics and software to open and close arms and deliver rewards (z-basic, Elba corp., Beaverton, OR, USA). Initially, rats were trained to alternate reward arms for 10 trials (i.e., access was allowed for a single alternating arm per trial). Then rats were given 10 free choice trials (i.e., simultaneous access to both arms) in order to determine if rats had a strong choice bias. No rats tested displayed a strong choice bias across days. Once animals completed the initial training session in less than 30 min for two consecutive days, probabilistic reversal training began (4–8 days). The initial training arm was randomly selected for each rat. On the first day of training, animals were allowed to freely choose either arm with the correct arm resulting in a one pellet reward 80% of the time and the incorrect arm never resulting in reward. Either choice resulted in a 10 s inter-trial interval (ITI; rat was located at the goal area) to control for the time it took to consume the reward following a correct choice in which a pellet was delivered. Rats then returned to the stem of the *T*-maze via a return track. Animals continued to choose either arm until it chose the correct arm 10 times in a row. The animal was then removed from the maze. The following day, the opposite arm was designated the correct arm and animals were required to reverse their choice in order to receive reinforcement. Again animals were allowed to freely choose either arm until 10 consecutive correct choices were made at which time they were removed and returned to the colony. For every subsequent day, the initially correct arm was psudorandomly chosen and was switched whenever a rat made 10 consecutive correct choices. A session continued for 2 h or until 200 trials were completed. Animals were not tested with inactivation or control injection until they were able to complete at least two reversals and 200 trials for two consecutive days. Once testing began, animals were randomly assigned to receive local LHb infusion of either saline injection or injection of baclofen/muscimol 6 min prior to the test beginning followed by the opposite treatment the following day in a repeated measures design. Animals performance was examined for any effects of order of treatment.

One possibility is that any results observed in experiment 2a are due to an inability of rats to learn discriminations in general, or an impaired ability to recall contingencies learned previously. In order to test these possibilities, an additional control study (experiment 2b) was performed in which the LHb was inactivated on either acquisition of an initial discrimination, or retention of that discrimination of the following day. Specifically, once animals were acclimated to the maze, rats (*n* = 20) were trained to receive 80% reinforcement on the arm opposite their innate bias observed during the maze acclimation stage. Once animals chose the correct arm 10 trials in a row, they were removed from the maze and returned to the colony. The following day, the opposite treatment (saline or baclofen and muscimol) was given and animals performed the same discrimination to 10 consecutive correct trials in the same manner as the previous day. In one group of animals LHb inactivation occurred during initial acquisition with saline treatment during retention trials while another group received the reverse injection schedule.

#### Stereotaxic Surgery

For experiment 1, recording tetrodes were constructed from 20 μm lacquer-coated tungsten wires (California Fine Wire). Tetrodes were places in custom made drives and impedances were measured at 1 kHz then, if necessary, gold-plated or replaced such that final impedances were 0.2–1.2 MΩ. In both experiments rats were deeply anesthetized with isoflurane, followed by administration of an antibiotic (Baytril, 5 mg/kg) and analgesic (Ketoprofen, 1 mg/kg). The skull was exposed and holes were stereotaxically drilled to allow for implantation of either recording electrodes (A-P: −3.5, M-L: ± 0.9, and D-V: 4–5 mm) or guide cannula (A-P: −3.5, M-L: ± 0.9, and D-V: 4.35 mm) dorsal to the LHb. Six animals in experiment 1 were implanted with a 6-tetrode, linear bundle drive unilaterally, and six animals with two 2-tetrode microdrives bilaterally. A reference electrode was also implanted near the anterior cortex (ventral to the brain surface 1–2 mm), and a ground screw was secured to the skull. The drives were then fixed to the skull with screws and acrylic cement. For experiments 2a and 2b, 31 rats were implanted with bilateral guide cannula (Plastics One, Roanoke, VA, USA) aimed 1 mm above the LHb (A-P: −3.5, M-L: ± 0.9, and D-V: −4.35 mm). Rats were allowed to recover for 5 days with free access to food and water. After recovery, rats were returned to a food restricted diet.

Experiment 1 rats were retrained until they completed 10 trials within an hour for two consecutive days. During retraining, tetrodes were slowly lowered to the LHb, no more than 320 μm/day. Once in the target region tetrodes were lowered in 40 μm increments in search of units, no more than 200 μm/day. Once a unit was found, recordings were conducted. At this point, experimental manipulations were also introduced in the behavioral task (see task description). Tetrodes were left in the same location for up to three sessions in an attempt to record units across multiple experimental conditions. For experiment 2, rats were placed on food restriction following recovery and then began training procedures.

#### Microinjection Procedure

A day before microinjection in experiments 2a and 2b, the injection cannula (Plastics One, Roanoke, VA, USA), which extended 1 mm beyond the guide was inserted into the guide cannula and left in place for 1 min. This was done to control for any initial mechanical damage done by the injector. On a test day, rats were injected with a combination of baclofen and muscimol (Bac/Mus, Sigma) in 0.9% saline, GABA b and a agonists respectively, or vehicle. Both injections used a volume of 0.2 μL (50 ng/0.2 μL baclofen and muscimol) and a 0.15 μL/min infusion rate. This is similar to other LHb inactivation studies that used baclofen and muscimol (Stopper and Floresco, 2014; Mathis et al., 2015). The injection cannula was connected to a 10 μl syringe (Hamilton) via polyethylene tubing (PE 20) using an infusion pump (KD Scientific).

#### Data Collection and Analysis

In experiment 1 all cellular recordings were conducted using a Cheetah data acquisition system (Neuralynx). Cell signals were filtered between 0.6 and 6 kHz, and digitized at 32 kHz. Neuronal spikes were recorded for 2 ms after a voltage deflection exceeded a predetermined threshold on any of the four channels of a tetrode (500–7000× amplification). Animal position data were sampled at 30 Hz via a ceiling mounted video camera that tracked LEDs attached to a preamplifier on the animal’s head. Signals were manually sorted using Offline Sorter (Plexon, Inc.) that allows segregation of spikes based on clustering parameters such as spike amplitude, spike duration, and waveform principle components. Cells were further analyzed if the waveform amplitude was at least 1.5 times that of the background cellular activity, and if the cluster boundaries were consistent across the session. The behavioral correlates of unit activity were analyzed using custom Matlab software (MathWorks Inc., Natick, MA, USA). Position data were used to manually place event flags to mark various aspects of behavior throughout the task including reward encounter, animal turns, inbound movement, trial starts, and errors. Given our hypothesis that the LHb regulates VTA dopamine cell responses to reward, reward-related responding in LHb cells were evaluated using similar methods that were used to identify VTA reward responses in prior studies (e.g., Jo et al., 2013; Puryear et al., 2010). In short, neural data were organized into peri-event histograms (PETHs) that were centered around the time of reward encounters (±2.5 s; 50 ms bins). Cells were considered to be reward related if peak (or valley) firing occurred within ±150 ms of reward encounters, and the mean firing rate of the ±150 ms window around the reward encounter was over 150% or under 75% of the mean session firing rate. Throughout the course of the experiment, it became clear that the LHb contained velocity correlated cells. Thus, firing rates of LHb neurons were correlated with the velocity of the animals as they traversed the maze. Based on animal tracking data, “instantaneous” velocity of the animal was determined by dividing the distance between two points by the inverse of the video sampling rate (Gill and Mizumori, 2006; Puryear et al., 2010; Mizumori et al., 2004). Each cell’s firing rate was then correlated with these velocity measures (Pearson’s linear correlation; α = 0.05) within the range of 1–30 cm/s. Velocity analysis did not include times when the animal was not moving, for example during reward consumption.

For local field potential analysis (LFP), signals from each tetrode within the LHb were analyzed in the following manner. Power was calculated using the multitaper Fourier analysis, mtspecgramc, from the Chronux toolbox (Mitra and Bokil, 2007; Bokil et al., 2010), using a 500 ms window with a 50 ms step. The resulting spectrogram was filtered for the theta frequency band (4–8 Hz) and binned relative to event timestamps. Values were interpolated where possible, otherwise they were set to NaN. The mean was taken over the theta frequency band using the MatLab function nanmean, which excludes NaN values. Finally, values were converted to dB using the relation 10*log10(μV^2^)/Hz and the mean was taken over the bins of each event occurrence, again using nanmean. Analysis of LFP velocity correlates matched that of unit analysis. Reward responses were analyzed by taking the 200 ms around reward encounter and comparing it with another 200 ms time window 1800 ms after the reward encounter which was a time found to have similar velocity to the reward encounter. Comparisons for significant changes around the reward encounter were tested using a student’s *t*-test with Bonferroni corrections for multiple comparisons. Proportions of responding LFP signals were analyzed for significant increases above chance with chi square tests. A two way analysis of variance (ANOVA) was used to test for differences around the time of reward between blocks one and two to measure whether reward manipulations change reward approach responses.

For experiment 2, an error analysis was conducted to determine whether inactivation caused changes in the ability to initially inhibit the previously correct choice pattern (perseverative errors) and/or the likelihood that an animal maintained the new choice pattern once selected and reinforced (regressive errors). The first trial of reversal learning was not counted as a perseverative error, but served as initial negative feedback. The following trials were divided into blocks of four trials. If a rat continued to choose the previous location in at least three of the four trials, the block was counted as perseveration. Once the rat made two correct choices in a given block, all subsequent errors were counted as regressive errors as in previous studies (Brown et al., 2012). Additionally, an analysis of win-stay and lose-shift probabilities was carried out. Win-stay probability is the likelihood that a rat will choose the correct arm if it was rewarded in the immediately preceding trial (the number of subsequent correct choices/the total number of preceding rewarded correct choices). Lose-shift probability is the frequency with which the rat shifted to the other choice when the correct arm was not rewarded on the previous trial (the number of subsequent incorrect choices/the total number of preceding unrewarded correct choices). This was observed on only a minority of trials. These measures are thought to represent sensitivity to positive and negative reinforcement respectively (Means and Holsten, 1992; Bari et al., 2010; Amodeo et al., 2012). The effects of LHb inactivation were assessed in terms of the number of trials per reversal, total number of reversals completed, and all error measures; these parameters were analyzed using a repeated measures Student’s *t*-test. For experiment 2b, a two way ANOVA was used to test for order of treatments as well as stage of discrimination.

#### Histology

After the completion of all recording sessions, tetrode locations and cannula placements were verified with marking lesions. Rats were deeply anesthetized with 4% isoflurane, and each tetrode (Experiment 1) was marked by passing a 15 μA current through each tetrode wire for 15 s. The animals were then given an overdose of sodium pentobarbital and transcardially perfused with 0.9% saline and a 10% formaldehyde solution. Brains were stored in a 30% sucrose in 10% formalin solution at 4°C for 1 week. The brains were frozen, and then cut in coronal sections (45 μm) on a freezing microtome. The sections were mounted on gelatin-coated slides, stained with cresyl violet, and examined under light microscopy. Only cells verified to be recorded in LHb were included in the data analysis. In experiments 2a and 2b, only cannula placements within the LHb were included in the analysis. For experiment 1, the locations of recorded cells were determined using standard histological reconstruction methods.

## Results

### Histology

**Figure 3:**
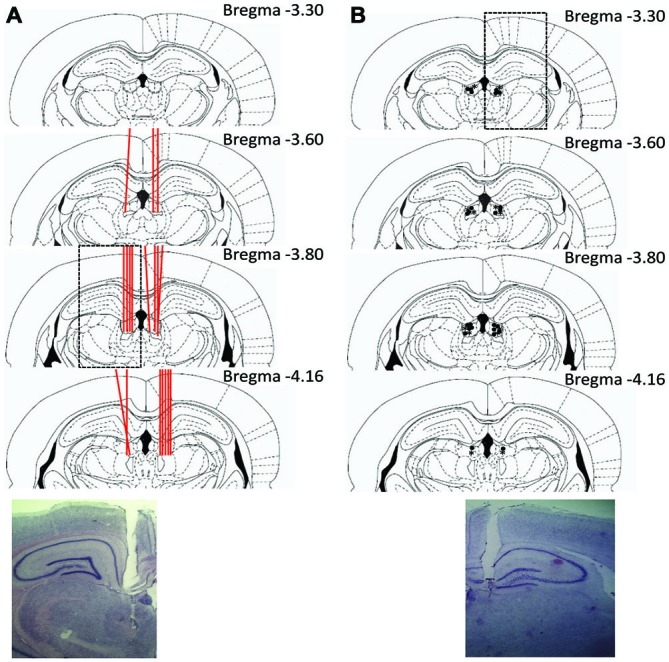
**Summary of histological results. (A)** Experiment 1 example section and schematic of tetrode placements within the LHb. Individual tetrode tracts are represented with red lines passing through the LHb. **(B)** Example and schematic of cannula placements for experiment 2. Injection cannula locations are represented with *’s for experiment 2a, and •’s for experiment 2b.

Histological results are summarized in Figure 3. Of the 12 animals implanted in experiment 1, LHb placed tetrodes were confirmed in six of these animals. In the LHb, a total of 36 unique units were recorded throughout this task. Many cells were recorded for multiple sessions (up to three) in attempt to capture their responses under various experimental manipulations. Units were considered the same cell if they were recorded at the same depth and had comparable waveforms. There was minimal ambiguity in this selection process with signals in the LHb being relatively sparse and often only one cell being recorded per session. Of the 31 rats implanted in experiment 2, 19 had bilateral cannula placements within the LHb that completed the study and were included in the analysis. Of the remaining rats, two did not meet training criteria during experiment 2a and were removed prior to injection. An additional rat had to be removed from the study due to complications following surgery. An additional two rats in experiment 2a had misplacements in the hippocampus (dorsal to the LHb) and completed six and three reversals respectively indicating no clear effect of a restricted hippocampal inactivation on the task. Of the seven rats with misplacements in experiment 2b, five rats had placements in the mediodorsal thalamus. Interestingly, in each of these cases, rats did not complete the task and qualitatively engaged in freezing behavior or a refusal to move. The remaining two anterior placements did not show any signs of impairment.

### Experiment 1

#### Behavior

Rats were exposed to one of three randomly chosen different reward manipulations during the second block of trials in a daily session. The total number of errors across both blocks of trials were compared. On sessions in which rewards were switched in the second block animals tended to make more errors in the second block (5.93 ± 1.14) than the first block (3.93 ± 0.68), however, this difference was not significant, *t*_14_ = 1.36, *p* > 0.05. Similarly, when rewards were omitted rats in the second block (4.77 ± 1.36) tended to make more errors than the first block (2.23 ± 0.63) which was not a significant difference, *t*_12_ = 1.45, *p* > 0.05. Finally, rats run in darkness during the second block (4.00 ± 1.49) were not significantly different from performance in the first trial block (3.00 ± 0.94), *t*_5_ = 0.47, *p* > 0.05.

#### Mean Firing Rates

**Figure 4:**
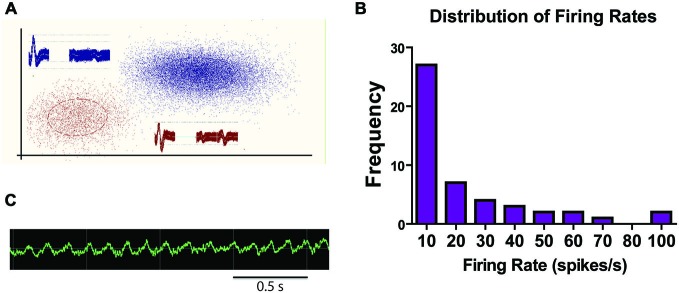
**Sample traces and summary of firing rates. (A)** Sample traces from a tetrode within the LHb with two distinct units. **(B)** Sample raw LFC signal from LHb during a recording session. **(C)** Frequency count of firing rate ranges for all recorded LHb units.

Sample LHb traces are shown in Figure 4A. Mean firing rates ranged from 0.5–107.6 spikes/s. Figure 4B shows the distribution of mean firing rates for LHb cells recorded in the study. Over half the sessions contained units with an average firing rate of less than 10 spikes/s. However, 44% of neurons had mean firing rates over 10 Hz. The wide range of average firing rates suggests that there were multiple cell types recorded throughout this study in accordance with other *in vivo* rodent or primate studies which have found population averages around 10 Hz or slightly below (Sharp et al., 2006; Matsumoto and Hikosaka, 2007; Aizawa et al., 2013; Goutagny et al., 2013).

#### Reward and Consumption: Single Unit Data

**Figure 5:**
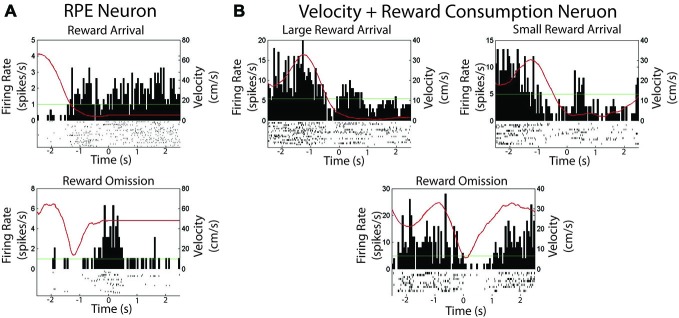
**Two neurons were found to be modulated by reward in experiment 1. (A)** Session summary of a neuron which met criteria for a reward prediction error neuron. Top: Firing rate on arrival at expected reward. Bottom: Firing rate on arrival at an unexpectedly omitted reward. **(B)** Session summary of neuron which correlated with velocity and reward consumption as shown when consuming a large or small reward or when reward is unexpectedly omitted. The green line is the session average firing rate for each neuron. The red line is the average velocity of the rat across the peri-event time histogram.

Only 2 (of 36) recorded cells showed firing that correlated with reward. Thus, here we provide only qualitative accounts for each cell. One neuron met criteria for a negative RPE cell; shown in Figure 5A. The cell was significantly inhibited at the time of reward encounter, and was excited when rewards are omitted (see “Materials and Methods” Section for criteria).

Another neuron was found to track both velocity and reward consumption, shown in Figure 5B. The cell was significantly correlated with velocity (Pearson’s *r* = 0.90, *p* < 0.001). It also exhibited firing when the animal was not moving, but consuming reward, with a qualitatively differential duration according to reward size. Excitation was not observed during reward omission, and the cell started firing only after the animal started to move.

#### Movement-Related Single Unit Responses

**Figure 6:**
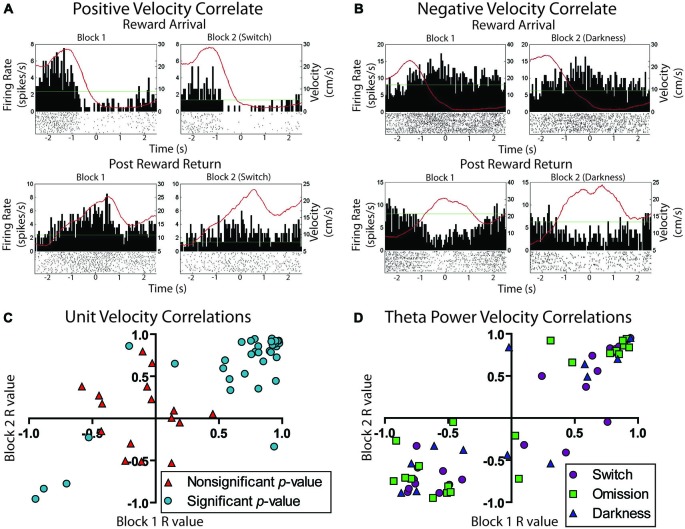
**Movement related activity in the lateral habenula. (A)** Example unit positively correlated with velocity at both reward arrival and return to the center platform. **(B)** Example unit negatively correlated with velocity under the same conditions. The green line is the session average firing rate for each neuron. The red line is the average velocity of the rat across the peri-event time histogram. **(C)** Scatterplot showing the stability of individual neuron movement correlations prior to and after reward manipulation. Cells are divided into those that showed an overall significant correlation with velocity during the session and those that did not show a significant correlation for the session. **(D)** LFP correlation of Theta power with velocity across blocks. Channels are divided into the three possible reward manipulations that were experienced by rats.

Overall, 66% of LHb cells (23/36) were significantly correlated with animal running speed. Of these running speed cells about half (12/23) showed positive correlations while the other half showed negative correlations (11/23). Example unit data are shown in Figures 6A,B. Figure 6C shows a scatterplot of the stability of these correlations between blocks. Units included in the plot were found to be significantly correlated with animal running speed across both trial blocks. Different colors/shapes indicate the experimental manipulation conducted during the session. No differences were observed for the number of positively, negatively, or uncorrelated with velocity cells across switch, omission, and darkness manipulations (*χ*^2^ = 0.39, *p* > 0.05, *χ*^2^ = 0.12, *p* > 0.05, and *χ*^2^ = 2.11, *p* > 0.05, respectively). In fact, many of these cells were recorded for multiple sessions and comparable correlations were found across experimental manipulations.

#### LFP Responses

**Figure 7:**
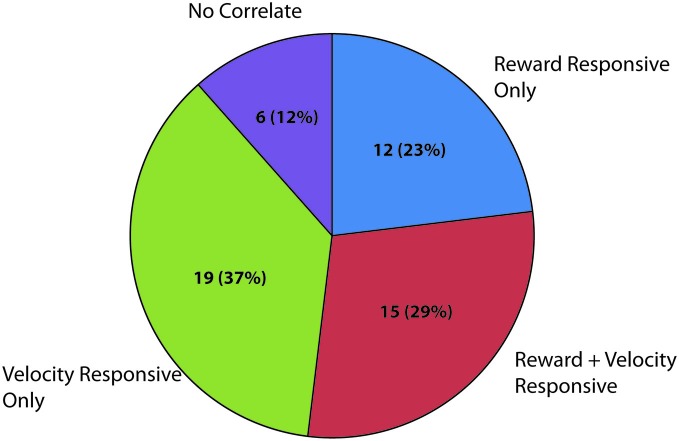
**Summary of significantly responding LFP channels during spatial navigation to reward.** Numbers represent significantly modulated channels in each category across sessions followed by the percentage of the total number of recorded LFP signals. Velocity responsive channels were defined as a significant correlation (Pearson’s linear) of instantaneous velocity with power in dBs in the theta band (4–8 Hz). Reward related channels were defined as channels which had significantly elevated theta power in the 200 ms surrounding reward arrival as compared with an equal sized time bin 1800 ms after reward encounter when similar velocities and positions were observed.

The theta frequency band (4–8 Hz) within the LFP data were analyzed for sessions containing units in the LHb (raw trace shown in Figure 4C). Fifty-two individual LFP signals were recorded across six rats. In general, theta power (dB) was found to be significantly correlated with animal running speed (velocity) as well as reward approach. Using instantaneous velocity correlations and modulation of theta power around the time of the reward, 46 of the 52 LFP signals were identified with either or both measures (Figure 7). Specifically, 12 were found to correlate with reward only, 15 with both reward and velocity, and another 21 with velocity only. Chi square analysis revealed proportions to be significantly above chance, *χ*^2^ = 6.31, *p* < 0.05; *χ*^2^ = 9.67, *p* < 0.05; and *χ*^2^ = 17.76, *p* < 0.05 respectively.

**Figure 8:**
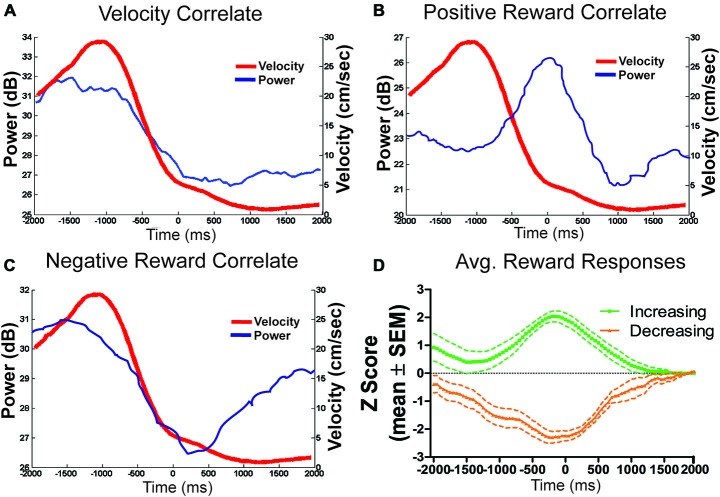
**Example theta power correlates and reward population responses. (A)** An example of LFP theta power which correlated only with velocity (*r* = 0.96). **(B)** An example of a positive reward approach only LFP theta channel. **(C)** Reward related signals were also equally likely to negatively respond to reward approach. **(D)** Shows the population average power converted to *z*-scores of all positively and negatively responding reward approach LFP theta signals.

LFP signals which significantly correlated with velocity (e.g., Figure 8A), were more likely to have positive (*n* = 32) correlates than negative (*n* = 4) correlates, *χ*^2^ = 12.83, *p* < 0.05. To examine the stability of velocity correlations, sessions were then grouped by tetrode location, such that a tetrode held at the same depth for multiple sessions would be considered a single “unit”. Grouped in this way, 18 out of 22 “units” were found significantly correlated with animals running speed. Figure 6D shows the stability of these correlations across blocks, indicating that LHb theta did not respond to reward or environmental changes. Specifically, from block 1 to block 2, only seven signals significantly changed their velocity correlations while 29 remained the same.

Of the 27 reward responsive signals, there was no difference in the proportions of positive (*n* = 13) and negative (*n* = 14) correlated signals, *χ*^2^ = 0.07, *p* > 0.05 (see Figures 8B–D). Further, the magnitude of response for both the positive and negative responses did not change across conditions from the first to second block indicating that the location of the reward or approach to it may have determined the LFP reward response rather than the size or presence of the reward itself. Specifically, an effect of time (±200 ms) around the reward was observed (*F*_4,200_ = 4.33, *p* < 0.05) but not of block (*F*_1,200_ = 0.19, *p* > 0.05) or an interaction, *F*_4,200_ = 0.81, *p* > 0.05.

### Experiment 2a

**Figure 9:**
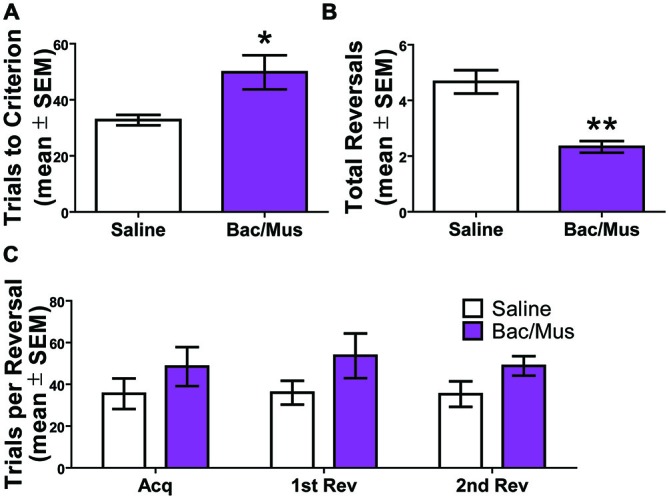
**Results of inactivation of the LHb during repeated probabilistic reversal learning. (A)** Inactivation of the LHb caused a significant increase on the trials to criterion for the initial acquisition of the test day. **(B)** There was also a decrease in the total number of completed reversals following LHb inactivation. **(C)** LHb inactivation resulted in a higher number of trials to criterion across competed discriminations. *p < 0.05, ^**^p < 0.01.

Inactivation of the LHb during repeated probabilistic reversal learning was performed in order to test the specific contributions of the LHb to behavioral flexibility performance as manifest through analysis of errors committed during performance. Six rats with bilateral good cannula placements were included in the final analysis. As shown in Figures 9A,B, comparison of inactivation (48.5 ± 9.3) with saline control injections (35.5 ± 7.4) revealed that inactivation of the LHb resulted in an increase in trials to criterion for initial acquisition (*t*_5_ = 4.01, *p* < 0.05), and a decrease in the number of reversals completed over the 200 trial session, *t*_5_ = 7.00, *p* < 0.01 (2.3 ± 0.2 and 4.7 ± 0.4, respectively). Additionally, as revealed in Figure 9C, the number of trials to complete a given discrimination did not differ across acquisition or any reversal for either saline or Bac/Mus treatment. Rather, LHb inactivation resulted in a consistently higher number of trials to criterion across discrimination stages, an effect of treatment (*F*_1,15_ = 11.24, *p* < 0.05), no effect of discrimination stage (*F*_2,15_ = 0.06, *p* > 0.05), and no interaction effect (*F*_2,15_ = 0.11, *p* > 0.05).

No effect of order of treatment was observed on total number of reversals completed (saline day one (4.7 ± 0.3) vs. saline day two (4.7 ± 0.9), *t*_4_ = 0.00, *p* > 0.05) and (Bac/Mus day one (2.3 ± 0.3) vs. Bac/Mus day two (2.3 ± 0.3), *t*_4_ = 0.00, *p* > 0.05) so the treatments were collapsed into single groups of saline and Bac/Mus for further analysis. Overall, no differences were observed between saline (2712.0 ± 183.3) and Bac/Mus (3274.0 ± 702.8) treatment in terms of the total time it took animals to complete the task, (*t*_5_ = 0.65, *p* > 0.05), revealing no gross changes in motor or sensory activity during the task.

**Figure 10:**
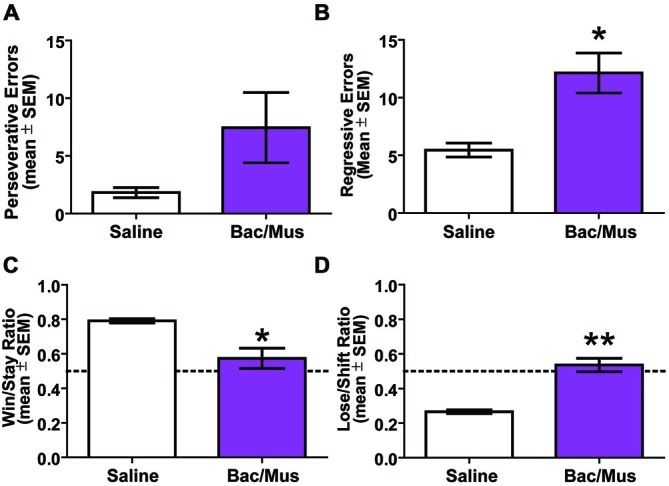
**Results of error analysis during repeated probabilistic reversal learning. (A)** LHb inactivation did not cause a significant increase in perseveration during serial reversals. **(B)** Regressive errors were significantly increased following LHb inactivation. **(C)** LHb inactivation caused a significant decrease in win-stay ratio. **(D)** There was also a significant increase in lose-shift ratio following LHb inactivation. The dashed line represents chance choices. *p < 0.05, ^**^p < 0.01.

An analysis of errors was conducted to further probe the deficit in the probabilistic reversal learning task following LHb inactivation (Figure 10). The deficit in discrimination performance was due to an increase across multiple error types. Specifically, no increase in perseverative errors was observed following Bac/Mus treatment (7.4 ± 3.0) compared with saline treatment (1.8 ± 0.4), *t*_5_ = 1.87, *p* > 0.05. There was an increase in regressive errors (*saline* = 5.5 ± 0.6 vs. *Bac/Mus* = 12.1 ± 1.7), *t*_5_ = 3.41, *p* < 0.05. To assess the sensitivity of LHb inactivation in relation to reward feedback, win-stay and lose-shift ratios were also analyzed. LHb inactivation led to a decrease in the win-stay ratio (*saline* = 0.79 ± 0.01, *Bac/Mus* = 0.57 ± 0.06, *t*_5_ = 4.01, *p* < 0.05) and in increase in the lose-shift ratio (*saline* = 0.27 ± 0.01, *Bac/Mus* = 0.54 ± 0.04, *t*_5_ = 5.77, *p* < 0.01).

### Experiment 2b

**Figure 11:**
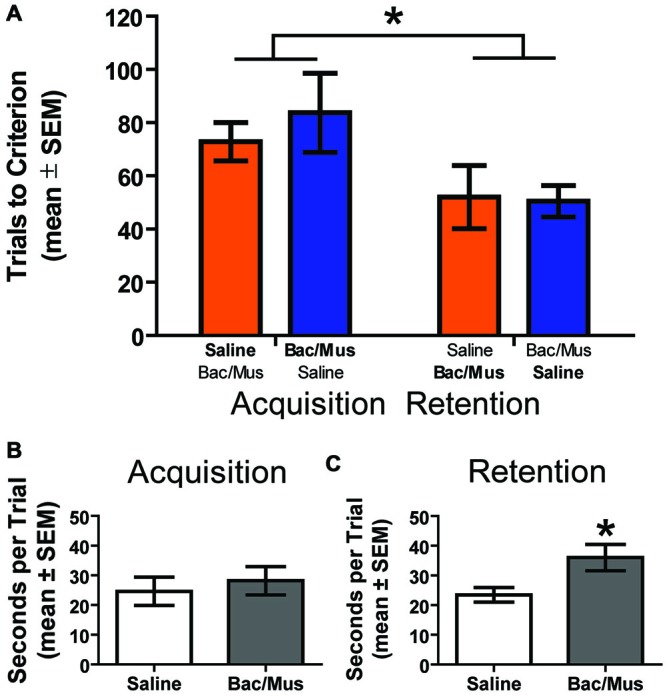
**Results of LHb inactivation on the acquisition and retention of an initial probabilistic discrimination. (A)** Results of a two way ANOVA revealed that treatment order did not affect the number of trials to criterion on either acquisition or retention of the probabilistic place/response discrimination. However, rats required fewer trials to reach criterion on retention than on acquisition. **(B)** No effect on the seconds per trial was observed on acquisition following LHb inactivation. **(C)** However, inactivation of the LHb during retention significantly increased the seconds per trial. *p < 0.05.

Due to an effect of LHb inactivation on the first daily discrimination in experiment 2a, one possibility is that learning in general is affected by the manipulation and the effects are not due to the requirement to perform flexibility *per se*. In order to address this, an additional control experiment was run in order to test the effects of LHb inactivation on initial probabilistic learning in rats which had not been trained to perform flexibility prior to testing (Figure 11). Two groups of animals were run receiving either Bac/Mus on acquisition and saline on retention the following day (*n* = 7), or saline on acquisition and Bac/Mus during retention (*n* = 6). A two way ANOVA revealed a significant effect of discrimination stage (*F*_1,11_ = 6.32, *p* < 0.05) but no effect of either treatment order (*F*_1,11_ = 0.19, *p* > 0.05) or an interaction (*F*_1,11_ = 0.33, *p* > 0.05). Specifically, Bac/Mus (83.71 ± 14.85) and saline (72.83 ± 7.23) treated rats required a similar number of trials to reach acquisition criterion as well as during retention (*saline* = 50.43 ± 5.87, *Bac/Mus* = 52.00 ± 11.82). However, overall acquisition (78.69 ± 8.14) took significantly more trials to criterion than retention (51.15 ± 5.78). Additionally no differences in the seconds per trial completed (*saline* = 24.60 ± 4.79, *Bac/Mus* = 28.19 ± 4.76, *t*_11_ = 0.53, *p* > 0.05) during acquisition were observed. However, during retention the seconds per trial completed after LHb inactivation (36.03 ± 4.46) was significantly higher than under control (23.48 ± 2.45) conditions, *t*_11_ = 2.57, *p* < 0.05 (Figure 11C). This change in the time per trial is in contrast with effects of inactivation on this measure across both experiments 2a and 2b where otherwise no difference was observed.

## Discussion

This study reveals for the first time that the LHb is involved when animals are required to express learned flexible behavior. Experiment 1 revealed that a majority of neurons in the LHb track ongoing movement, with often high correlations (>0.9) with running speed. The population of movement correlates is split, with half these cells being positive correlates and half negative. In addition, an analysis of theta rhythms recorded simultaneously with LHb unit activity revealed that running speed information is also represented at the population level. Additionally, the approach to the reward area also resulted in a significant increase in theta power in 52% of the recorded LFP signals. Neither theta reward nor velocity power correlates changed when reward contingences were manipulated in the second block during, reversal of reward placements, omission of reward, or in darkness. This pattern of effects suggests that the tracking of movement and reward approach by these cells/population signals are more related to ongoing behavior and not tied to reward-specific responses during the task. In contrast, only 2 out of 36 unique units recorded were found to be reward related—one being linked to consummatory behavior in addition to velocity, and the other exhibiting activity suggestive of a code for an RPE.

In experiment 2a, LHb inactivation during repeated probabilistic reversals of a spatial/response task resulted in impairment in performance that was consistent across multiple reversals, and not related to nonspecific sensory or motor impairment. Animals required more trials to criteria per completed discrimination, suggesting a general impairment in reward discrimination learning when animals need to be flexible in their behavior. Additionally, an increase in regressive errors was observed along with changes in win-stay and lose-shift ratios which suggest a reduction in both reward and non-reward sensitivity. In experiment 2b, however, no effects of LHb inactivation were observed on either the initial acquisition of a probabilistic reward discrimination or on the retention of that discrimination on the following day. Overall, the combined results of these experiments indicate that the LHb tracks ongoing behavioral information for the purpose of facilitating processes that are required for behavioral flexibility. Specifically, LHb may track ongoing behavior so that actions toward goals are optimized. For this reason the LHb may only be required when behavioral strategies must be used in cognitively demanding tasks in order to track ongoing information about specific motor behaviors currently being performed. Specifically, in non-aversive situations, LHb may be involved to the extent that behavioral response strategies are needed to achieve a goal; it may track and direct behaviors to follow dynamic goal information.

The general contribution of the LHb to expressing learned goal directed behavioral activity under demanding conditions is supported by related findings. Mathis et al. (2015) found that when the LHb was inactivated or if excitatory transmission was blocked with the AMPA receptor antagonist CNQX, rats were unable to express a learned spatial memory of the escape platform location in the Morris water maze and instead showed thigmotaxis. Thigmotaxisis is a behavioral strategy initially used when rats are first placed into the maze, one that is characterized by a preference to remain close to the perimeter of an environment. The stress of the water maze may be sufficiently demanding in this instance to require the LHb. Inactivation of the LHb in well trained rats also disrupted both delay and probability discounting by inducing random patterns of choice which could be interpreted as a default or guessing mode (Stopper and Floresco, 2014). These studies may relate to other findings that LHb optogenetic activation can promote active, passive and conditioned behavioral avoidance which suggests that LHb activity is important for learning specific behaviors in response to external/internal stimuli (Stamatakis and Stuber, 2012).

The role for the LHb in utilizing context or behavioral state information to guide behavior can be further illuminated by comparing it with a recent proposal for basolateral amygdala function put forth by Wassum and Izquierdo (2015). The authors propose that the role of the basolateral amygdala is to assign an integrated value signal to specific stimuli in order to guide adaptive responses. We propose a more fundamental role for the LHb in behavioral flexibility in which it provides the current behavioral state of the animal in order to properly select the appropriate actions within that context. This difference is best exemplified by examining the effects of inactivation of these structures during discounting behaviors. As outlined above, inactivation of the LHb leads to a guessing mode during delay and probability discounting where each choice is selected roughly half of the time (Stopper and Floresco, 2014). Inactivation or DA manipulation of the basolateral amygdala in both delay and probability discounting causes rats to prefer the smaller certain or immediate reward once either the probability decreases or the delay increases (Winstanley et al., 2004; Churchwell et al., 2009; Ghods-Sharifi et al., 2009; Larkin et al., 2015). Crucially, however, discounting curves in both tasks increased rather than decreased in slope indicating a change in preference rather than entering a guessing mode. This helps to distinguish the proposed role for the LHb in identifying the current behavioral state of the animal from a role in valuation of specific actions as is suggested for the basolateral amygdala (Wassum and Izquierdo, 2015).

The mechanism by which neural signals within the LHb translate to an ability to switch behaviors under cognitively demanding conditions in freely moving rodents remains somewhat uncertain. One possible mechanism that has been proposed is through signaling RPEs as has been seen with LHb neurons in head-fixed monkeys (Matsumoto and Hikosaka, 2007; Proulx et al., 2014; Stopper et al., 2014). Although it is exciting to confirm the presence of RPEs in LHb cells, this is a considerably smaller proportion of cells than expected, as the Hikosaka had found over 80% of primate LHb cell activity to be related to rewards (Matsumoto and Hikosaka, 2007). Considering the differences in the animals and task used, there are a number of reasons why this could be the case. The original task used by Matsumoto and Hikosaka was much more Pavlovian in nature compared to our maze based tasks. While LHb neurons did show some excitation during unrewarded trials, these neurons showed much greater responses to the cues that predict reward omissions. They also showed high levels of responding during the first trial where a reward was omitted; however, once the animal knows whether or not it will be rewarded and the outcome is congruous with the expectation, there is little change from baseline at the actual outcome. Given that the animals in experiment 1 were highly trained, this may explain why we failed to observe more responses directly at the time of reward. However, this cannot completely account for the lack of observed RPE signals as in every case, the rat could not predict which rewards would be switched or omitted during a given session. In addition, the task used in primates requires the subject to be head-fixed, which would abolish movement related neuronal activity. It could be argued that in the present study, if the rat LHb was tracking some sort of discrete cue, movement-related activity of the LHb cells somehow masked these signals. This is not particularly probable, as the task was designed without explicit cues. However, it may be the case that movement itself is the most reliable cue for when rewards will be received.

In our task, the animal is very well trained, and although navigation is goal directed, some aspects of the task are highly predictable and reliable. For example, all rewards are the same distance away from the center; therefore, once a choice is made, animal trajectory becomes perhaps the most reliable reward predictor. Movement correlates in rat LHb have been previously reported during a pellet-chasing task, which encourages the animals to run in semi random trajectories (Sharp et al., 2006). They found that ~10% of recorded neurons to be significantly correlated with running speed as compared to our 66% of neurons and LFP theta signals. If the LHb is tracking reward cues, animal movement may be overrepresented in this task. This would suggest that movement itself can serve as a reward predictive stimulus in freely moving animals. This is supported by the finding that despite only finding 2 of 36 neurons related to reward consumption, over half of the LFP signals recorded showed in increase in theta power during reward approach that was not related to consumption. Theta power synchrony is thought to be an effective means for relaying information between brain areas (Panzeri et al., 1999; Fries, 2005) and has been found to be related to behavioral performance between the LHb and the hippocampus (Goutagny et al., 2013). This raises the possibility that the LHb may also use theta synchrony to relay velocity/reward approach information to other areas as well such as the VTA (Kim et al., 2012). Indeed, velocity or reward related approach neural correlates have been observed in the radial arm spatial memory task previously suggesting this information may be important for reward learning (Puryear et al., 2010). A similar interpretation for strong velocity correlated neural activity has been proposed for another major afferent system of VTA DA neurons, the lateral dorsal tegmentum. The latter neurons were postulated to regulate reward responses of DA neurons according to the learned behaviors needed to obtain rewards (Redila et al., 2015).

To dissociate predictive movement from movement *per se*, future studies should include an open field component. If a proportion of the movement correlates found in the present study were actually reward predicting cues, then a subpopulation of these cells would not exhibit velocity correlates if recorded during general ambulation. To further investigate reward related responses, future studies should also consider using a task featuring explicit cues for rewards in order to observe LHb responses to reward predicting cues. Regardless of the interpretation of the movement correlates, it is clear that a proportion of LHb cells are heavily modulated by ongoing speed of the animal in freely navigating rats. LHb cells recorded in this study showed either positive or negative running speed correlates, which suggests that there may be subpopulations that code for different movement parameters. Either type of velocity code could inform other structures and/or other LHb cells of ongoing behavior in anticipation of reward encounters. If the primary function of the LHb is to suppress movement during unfavorable conditions, as Hikosaka (2010) proposes, it would be adaptive for movement suppression networks to be informed of ongoing behavior. In this way, velocity information could bias action specific learning, and discourage actions that lead to negative outcomes. Velocity information could also be helpful for more specific control over movement suppression such as the timing of the suppression.

Experiment 2 showed a deficit in behavioral flexibility following inactivation. One possible cause of this effect is the connection of the LHb to the hippocampus. The hippocampus has been proposed to signal context and context changes important in adaptive decision making (Mizumori et al., 2004; Smith and Mizumori, 2006; Kim and Frank, 2009; Bachevalier et al., 2015). LHb cells have been found to be more active and to phase lock during hippocampal theta than during slow wave sleep across the sleep wake cycle (Goutagny et al., 2013). Theta generated within the LHb was also highly synchronized with hippocampal theta, and this synchrony was linearly correlated with performance on a memory task. The hippocampus is thought to be important for reversing probabilistically learned tasks in humans (Shohamy et al., 2009; Dickerson et al., 2011; Delgado and Dickerson, 2012). This effect is not due to any spatial aspects of the task which suggests that the hippocampus is important for applying higher order signals to goal directed behavior such as would be necessary when reward contingencies change probabilistically. This could account for the effects observed in experiment 2 although this is largely speculative. Support for this account of a loss of context however, can be found in the fact that in the present study, animals’ win-stay and lose-shift ratios fell to around 50% suggesting they are likely guessing. This phenomenon has also been seen in both delay and probability discounting following LHb inactivation (Stopper and Floresco, 2014). In experiment 1 theta power was correlated with velocity which also supports that this velocity related information may serve as a predictive stimulus during behavioral flexibility through cross talk with context related signals in the hippocampus. Further research should determine if this interaction could account for deficits observed following LHb interaction. How the LHb, hippocampus, and midbrain monoaminergic systems interact during behavioral flexibility clearly requires more research.

Ten subnuclei have been described in the LHb (Geisler et al., 2003), but their behavioral relevance has not been studied. In the present study, although each area was not systematically examined due to the limitations of using a movable microdrive to record signals, no differences between either LFP or single units recorded medially or laterally were observed. This suggests that the information broadcast by the LHb at least in the case of appetitively driven behavioral flexibility, is more or less uniform and likely additional input into target structures is needed in order to achieve any required signal specificity. This view is supported by the general effect observed in experiment 2a in which a broad increase in both trials to criterion as well as error types was observed. More specifically, results support that the LHb contributes to behavioral flexibility by regulating action selection rather than a more specific influence on changing behavior in response to positive or negative reinforcement given that both win-stay and lose-shift behavior fell to nearly chance levels. Understanding responses of the LHb in a wide variety of tasks as well as using more targeted recording techniques is necessary to elaborate both the types of behavior it is involved in as well as whether signal specificity exists within the LHb as the anatomy might suggest.

Based on several recent technological advances, we feel that the LHb is on the cusp of giving up many of the secrets about its underlying behavioral and physiological functions. Proulx et al. (2014) have outlined how optogenetics combined with transgenic Cre-driver mouse lines promises a new understanding of how various sub regions and their respective afferent and efferent connections contribute to LHb behaviors. Other recent work sought to understand precisely how the LHb contributes to monoamine output during behavior and is ongoing (Shen et al., 2012; Stamatakis and Stuber, 2012; Stopper et al., 2014). The present results further suggest that movement of the animal during behavior should also be taken into account when addressing the role of the LHb in learning and memory functions. Due to the discovery of synchrony between the hippocampus and the LHb during memory related tasks (Goutagny et al., 2013), the wealth of information known about hippocampal function in learning and memory as well as behavioral flexibility especially in relation to spatial information offers a promising means of examining LHb contributions to well delineated cognitive systems. By combining the latest functional neuroanatomical techniques with a range of complex behavioral tasks, including those examining behavioral flexibility, the possibility to answer some very longstanding fundamental questions about LHb functions is within reach (Sutherland, 1982).

Simultaneous with the recent upsurge of neuroscience research on LHb function, it has become clear that this region is of interest for its relevance in multiple psychiatric disorders including addiction, depression (Lecca et al., 2014; Proulx et al., 2014), and to a lesser extent, aspects of bipolar disorder (Savitz et al., 2011), schizophrenia (Shepard et al., 2006), and Parkinson’s Disease (Luo et al., 2015). This is primarily due to LHb connectivity between the limbic forebrain and dopaminergic and serotonergic systems, which are strongly associated with these disorders. Common to these diseases is also an impairment in behavioral flexibility (Berman et al., 1986; Morice, 1990; Koerts et al., 2009; Dickstein et al., 2010; Walshaw et al., 2010; Nesic et al., 2011; van Holst and Schilt, 2011). Indeed interventions such as deep brain stimulation of the LHb have already been performed on patients with promising results (Sartorius et al., 2010). However, it is heretofore unknown whether behavioral flexibility in this treatment is also affected. Regardless, the fact that LHb has been connected with pathological mood states as well as other maladaptive changes in behavior makes it likely that these conditions interact with the presently proposed role of the LHb in signaling current behavioral state to organize action selection. Support for this interaction comes from findings that rodent models of depression as well as antidepressant treatment leads to changes in both the input to and output from the LHb (Shabel et al., 2014). However, how these changes might affect the current findings of largely velocity related neural firing and reward approach theta oscillations remains unknown. This serves to highlight that a deeper understanding of the LHb is essential for more refined therapies.

## Conflict of Interest Statement

The authors declare that the research was conducted in the absence of any commercial or financial relationships that could be construed as a potential conflict of interest.
